# What makes written words so special to the brain?

**DOI:** 10.3389/fnhum.2014.00634

**Published:** 2014-08-22

**Authors:** Mohamed L. Seghier, Urs Maurer, Gui Xue

**Affiliations:** ^1^Wellcome Trust Centre for Neuroimaging, Institute of Neurology, University College LondonLondon, UK; ^2^Department of Psychology, University of ZurichZurich, Switzerland; ^3^National Key Laboratory of Cognitive Neuroscience and Learning & IDG/McGovern Institute for Brain Research, Beijing Normal UniversityBeijing, China

**Keywords:** reading, word processing, learning, multilingualism, dyslexia, laterality, fMRI, ERP

Reading is an integral part of life in today's information-driven societies. How the human brain sees and processes words is the focus of this ebook. It includes a collection of 22 papers that illustrate current issues in the neurobiology and psychophysics of word processing. Using varieties of behavioral tests and neuroimaging techniques, they investigated word processing mechanisms across different alphabetic and logographic writing systems, such as English, Chinese, Arabic, Japanese, French or German. Each paper provides useful literature reviews, methodological developments and a host of novel findings that will inspire future investigations into the neural systems that support reading.

Several behavioral, fMRI and ERP studies investigated how word-likeness modulated the underlying cognitive and neural processing. The fMRI studies focused on many regions of the reading system, in particular a region in the left ventral occipitotemporal cortex known as the visual word form area (VWFA), whereas the ERP studies featured prominently the N1/N170 component around 200 ms post-stimulus. Participants were mainly healthy skilled readers, although a few studies have also looked at reading in dyslexic, autistic, or congenitally deaf subjects.

Ludersdorfer et al. (2013) investigated how the VWFA responds to visual and auditory stimuli that differed in their word-likeness. VWFA activation decreased for visual stimuli from false-fonts over pseudowords to words, presumably reflecting more efficient processing of familiar words. In contrast, auditory stimuli lead to a general deactivation in visual areas for all stimuli, except for the VWFA where deactivation was spared for word and pseudoword stimuli, presumably due to modulation by linguistic information. Deng et al. (2013) used two cross-modal tasks, phonological retrieval of visual words and orthographic retrieval of auditory words, to examine the unimodal and multimodal regions for logographic language processing. The VWFA responded exclusively to visual inputs, whereas an adjacent region in the left inferior temporal gyrus showed comparable activation for both visual and auditory inputs. A role of the VWFA in integrating visual and auditory processes is also suggested by McNorgan et al. (2013) who reported correlations between a behavioral phonemic awareness task (phoneme elision) and neural activation in an audiovisual condition in typically developing children. No such correlations were found in children with reading disability, nor in any of the groups for unimodal stimuli.

Using ERP, an inverse word-like effect was also found for single letters by Herdman and Takai (2013), who reported an increased and delayed N1 for pseudoletters than letters, which was not modulated by attention. As studies using entire words typically report a reversed effect, this may suggest that processing single letters differs from processing letter strings and that early orthographic processing of letters is largely automatic. Hasko et al. (2013) not only showed a positive word-like effect in the N1 component, as the N1 was larger for letter than false font strings in normally reading children, but they also revealed that this effect was reduced in dyslexic children, presumably reflecting deficient orthographic processing in dyslexics. While Hasko et al. (2013) did not find N1 differences between pseudohomophones and words in children, Taha and Khateb (2013) found a larger N1 for pseudohomophones than words in Arabic, suggesting that such effect may depend on reading development, task, or properties of the writing system.

Orthographic analysis in the later part of the N1 also seems to be sensitive to stimulus repetition, as shown by Du et al. (2013). However, such N200 repetition effects appear to be delayed if word form configuration is changed, which can be achieved in Chinese by switching characters in two-morphemic words. Regarding the impact of orthographic depth on reading routes, Buetler et al. (2014) recorded electrical brain activity in highly proficient bilinguals who read the same pseudowords either in German or French. The topography of the ERPs to identical pseudowords differed 300–360 ms post-stimulus onset when the pseudowords were read in different orthographic depth context. Their findings suggest that reading in a shallow context relies more on non-lexical pathways with greater engagement of frontal phonological areas, whereas reading in a deep orthographic context recruits less non-lexical pathways with greater engagement of visuo-attentional parietal areas.

We note that many of these fMRI and ERP studies used conditions which differed in word-likeness, and reported either positive or negative relations in the observed neural activation. The reason for this divergence is still poorly understood, but probably reflects that orthographic processing includes both visual processing and modulation by linguistic information.

Regarding the elusive role of the VWFA, a review by Vogel et al. (2014) challenges current models that posit a functional specialization of the VWFA solely for words. They argue that the VWFA is not used specifically or even predominantly for reading. In their model, the VWFA is used in processing visually complex stimuli in “groups,” and it is strongly connected to the dorsal attention network so that attention can be directed to familiar stimuli, such as words, in groups. They suggest that the VWFA can be seen as a brain region with specific processing characteristics rather than a brain region devoted to a specific stimulus class. Given the strong interactions between the reading and the attentional system, Montani et al. (2014) conducted a behavioral study to examine the impact of spatial attention on written word perception. They found that high frequency word identification was best in the neutral cue condition when attention was directed to both the possible locations, whereas pseudowords (and a similar trend for low frequency words) were better identified in the valid cue condition when attention was focused on the target location.

Beside the ventral visual stream, modality-specific responses and interaction between orthography and linguistic components are also found elsewhere. Kollndorfer et al. (2013) used independent component analysis on fMRI data, collected during two language comprehension and production tasks with visual and auditory stimuli, and showed that the intraparietal sulcus and the hippocampus were predominately activated in the visual modality. In reading Chinese compound words, Zhan et al. (2013) found that mixed pseudohomophones, which shared the first constituent with the base words, were more difficult to reject than non-pseudohomophone non-words, and pure pseudohomophones, which shared no constituent with their base words. This effect was accompanied by increased activation of bilateral inferior frontal gyrus, left inferior parietal lobule, and left angular gyrus, and stronger effective connectivity of a phonological pathway from left inferior parietal lobule to left inferior frontal gyrus for the mixed pseudohomophones. Hillen et al. (2013) used the “Landolt” paradigm to dissociate linguistic and orthographic brain networks from those involved in occulomotor control and attention. In this paradigm, subjects were asked to scan for targets (Landolt's rings) in a reading-like fashion from left to right when all letters were replaced by closed circles. Significant fMRI activations were identified in right superior parietal cortex and postcentral gyrus, most likely related to gaze-orienting, which suggests the usefulness of the “Landolt” paradigm in dissociating linguistic from non-linguistic factors during reading.

To depict the interactions between semantic and phonological processing areas, Boukrina and Graves (2013) assessed effective connectivity while participants read aloud words of high or low spelling-sound consistency, word frequency, and imageability. Semantic areas significantly interacted with phonological areas, and connectivity patterns depended on word properties. Interestingly, they found that modulation of the inferior temporal and angular gyri connectivity correlated with reading performance. Some of the connectivity patterns were better predicted by the connectionist than the dual-route cascaded model. Regarding the role of subcortical structures in reading, Oberhuber et al. (2013) found that the putamen was mainly involved in articulating speech during reading, as compared to picture and color naming. Intriguingly, pseudowords showed greater activation in the anterior putamen, which is consistent with the role of this subregion in the initiation of novel sequences of movements. In contrast, words showed greater activation in the posterior putamen, which is consistent with studies that associated this putaminal subregion with memory guided movement.

To investigate the effect of language experience on reading, Li et al. (2014) examined differences in brain activation between Chinese congenitally deaf individuals and hearing controls during character reading. They found that congenitally deaf individuals showed less activation than controls in left inferior frontal gyrus, but greater activation in several right hemisphere regions including inferior frontal gyrus, angular gyrus, and inferior temporal gyrus, and the deaf individuals who are fluent readers showed less activity in the right hemisphere. Regarding reading in other clinical populations, Moseley et al. (2013) used fMRI to investigate semantic deficits in adults with autism spectrum conditions. They found that, compared to typically developing controls, the high-functioning adults with autism showed a deficit in semantic processing of action-related words, which, intriguingly, significantly correlated with the hypoactivity of motor cortex to these items.

Another interesting topic is how lateralized reading processes interact with task and script. In a behavioral study, Perrone-Bertolotti et al. (2013) investigated hemispheric specialization and inter-hemispheric interactions during a lexical decision task within a divided visual field presentation of verbal material. The authors manipulated three types of information (i.e., perceptual, semantic, and decisional) to determine how the type of information modulates inter-hemispheric cooperation. Their findings suggest inter-hemispheric cooperation is less likely to emerge during pre-lexical (perceptual) and/or post-lexical (decision-making) processing, but mainly occurred during lexical semantic processing when the semantic information was shared between hemispheres. Koyama et al. (2014) tested whether left-lateralized fMRI activations for reading differ between first (L1) and second (L2) languages in bilingual L2 readers. They asked late L2 learners to perform a visual one-back matching task either in English or Japanese. Weaker left lateralization was observed in the posterior lateral occipital region for logographic Kanji compared with syllabic (Kana) and alphabetic (English) scripts. When both L1 and L2 scripts were non-logographic, functional lateralization did not differ between L1 and L2 scripts in any region. Remarkably, they showed that functional lateralization for L2 visual word processing predicted L2 reading competency.

As most of the above studies focused on single word learning, little is known about the neural correlates of text comprehension. To address this issue, Swett et al. (2013) reported that, compared to single word comprehension, left posterior cingulate cortex and left angular gyrus were activated only for discourse-level comprehension. Over the course of comprehension, reliance on the same regions in the semantic control network increased, while a region in intraparietal sulcus associated with attention decreased. In addition, central ideas are functionally distinct from peripheral ideas, showing greater activation in the posterior cingulate cortex and precuneus.

Last but not least, two papers used psychophysics and mathematical modeling to characterize other word properties. Starrfelt et al. (2013) explored the word superiority effect, which refers to the observation that when written stimuli are degraded by noise or brief presentation, letters in words are reported more accurately than single letters and letters embedded in non-words. With a novel combination of psychophysics and mathematical modeling, they showed that word superiority is due to single words being simply processed faster than single letters (at least for simple short words). However, there is a limit to this effect as letters are perceived more easily than words in particular when multiple stimuli are presented simultaneously. For neuroimaging studies interested in the impact of spatial frequencies upon brain responses along the different reading pathways, Melmer et al. (2013) introduced some Fourier spectrum based measures that are useful for assessing statistical image properties. They showed how those statistical properties can reflect more global aspects of text, including for instance its aesthetic appeal. Their findings suggested that the statistical properties of different categories (regular text, aesthetic writing, calligraphy, ornamental art) were similar across cultures.

Overall, the papers in this ebook illustrate the wide range of techniques that can be used to reveal the functional anatomy and the time course of activity within the reading system. The exciting new insights that emerged from those studies can deepen our understanding of the mechanisms of individual differences in learning to read, and may help to guide the discovery of novel diagnostic tools and biomarkers for reading disorders.

## Conflict of interest statement

The authors declare that the research was conducted in the absence of any commercial or financial relationships that could be construed as a potential conflict of interest.
